# Language Patterns Discriminate Mild Depression From Normal Sadness and Euthymic State

**DOI:** 10.3389/fpsyt.2018.00105

**Published:** 2018-04-10

**Authors:** Daria Smirnova, Paul Cumming, Elena Sloeva, Natalia Kuvshinova, Dmitry Romanov, Gennadii Nosachev

**Affiliations:** ^1^Department of Psychiatry, Addictology, Psychotherapy and Clinical Psychology, Samara State Medical University, Samara, Russia; ^2^Centre for Clinical Research in Neuropsychiatry, University of Western Australia, Perth, WA, Australia; ^3^School of Psychology and Counselling, Queensland University of Technology, QIMR Berghofer Institute, Brisbane, QLD, Australia; ^4^Department of Pedagogy, Psychology and Psycholinguistics, Samara State Medical University, Samara, Russia

**Keywords:** euthymic state, language patterns, mild depression, negative pronouns, normal sadness, past tense verbs, personal pronouns, word use

## Abstract

**Objectives:**

Deviations from typical word use have been previously reported in clinical depression, but language patterns of mild depression (MD), as distinct from normal sadness (NS) and euthymic state, are unknown. In this study, we aimed to apply the linguistic approach as an additional diagnostic key for understanding clinical variability along the continuum of affective states.

**Methods:**

We studied 402 written reports from 124 Russian-speaking patients and 77 healthy controls (HC), including 35 cases of NS, using hand-coding procedures. The focus of our psycholinguistic methods was on lexico-semantic [e.g., rhetorical figures (metaphors, similes)], syntactic [e.g., predominant sentence type (single-clause and multi-clause)], and lexico-grammatical [e.g., pronouns (indefinite, personal)] variables. Statistical evaluations included Cohen’s kappa for inter-rater reliability measures, a non-parametric approach (Mann–Whitney *U*-test and Pearson chi-square test), one-way ANOVA for between-group differences, Spearman’s and point-biserial correlations to analyze relationships between linguistic and gender variables, discriminant analysis (Wilks’ λ) of linguistic variables in relation to the affective diagnostic types, all using SPSS-22 (significant, *p* < 0.05).

**Results:**

In MD, as compared with healthy individuals, written responses were longer, demonstrated descriptive rather than analytic style, showed signs of spoken and figurative language, single-clause sentences domination over multi-clause, atypical word order, increased use of personal and indefinite pronouns, and verb use in continuous/imperfective and past tenses. In NS, as compared with HC, we found greater use of lexical repetitions, omission of words, and verbs in continuous and present tenses. MD was significantly differentiated from NS and euthymic state by linguistic variables [98.6%; Wilks’ λ(40) = 0.009; *p* < 0.001; *r* = 0.992]. The highest predictors in discrimination between MD, NS, and euthymic state groups were the variables of word order (typical/atypical) (*r* = −0.405), ellipses (omission of words) (*r* = 0.583), colloquialisms (informal words/phrases) (*r* = 0.534), verb tense (past/present/future) (*r* = −0.460), verbs form (continuous/perfect) (*r* = 0.345), amount of reflexive (e.g., myself)/personal (*r* = 0.344), and negative (e.g., nobody)/indefinite (*r* = 0.451) pronouns. The most significant between-group differences were observed in MD as compared with both NS and euthymic state.

**Conclusion:**

MD is characterized by patterns of atypical language use distinguishing depression from NS and euthymic state, which points to a potential role of linguistic indicators in diagnosing affective states.

## Introduction

Mild depression (MD) is a common mental state (1), observed in 15% of the adult population (2), with only 23% receiving any treatment (3). MD is mostly related to life stresses (4) and [unlike moderate and severe major depressive disorder (MDD)] is poorly responsive to antidepressant medication (1, 5, 6). Nonetheless, MD [as distinct from subthreshold, minor depression (7) or normal sadness (NS) (8, 9)] is a serious medical condition causing professional and personal disabilities (10–12). Indeed, MD is associated with unemployment in 16% of cases (13). The chronic course of mild depressive symptoms within dysthymia brings an elevated suicidality risk, compared with MDD (14). MD is often prodromal to MDD (7, 15, 16). NS in the absence of clinical depression is also frequent (29.8%) in the general population (17).

The ICD-10 (18) diagnosis of MD requires four symptoms, whereas the DSM-V (19) criteria are based on seven main symptoms, and the Hamilton Depression Rating Scale (HDRS) gives an MD diagnosis threshold for scores ranging from 7 to 17 as widely accepted by clinicians or cutoff scores from 8 to 16 as suggested by the recent severity classification of HDRS (20–23). However, depression is heterogeneous and presents with highly variable clinical symptoms, so its diagnosis cannot be made merely by the number of symptoms, but should include their detailed analysis and causal relations (24–26). Diagnosis of MD was reported to be less stable compared with diagnosis of severe depression using ICD-10 criteria and was characterized by a fair level of agreement (kappa = 0.25) between clinicians compared with the moderate reliability in severe depression cases (kappa = 0.53) (8, 27). The claimed high prevalence of MD is sometimes viewed with skepticism, given the questionable reliability of psychiatric diagnoses in general (28), and especially with respect to the differentiation of MD from NS (8, 29). Correct recognition of subthreshold forms of NS is based upon the number, duration, and quality of presented symptoms (30). Despite the elaboration of criteria cited above, psychiatry still lacks objective clinical tests of symptoms comparable with those routinely used in other medical disciplines (31). Affective (e.g., decreased mood) and cognitive (e.g., negative content of thoughts) components of MD and NS are mostly expressed through language, while more severe forms of depression are also recognized by a motor component (e.g., slow bodily movements). The search for objective indicators of MD vs. NS might help to increase the reliability of MD diagnosis. Andreasen and Pfohl (32) first showed that language is a specific marker of depression, and currently active study groups have concluded that an analysis of natural language processing could afford the foundation for developing objective diagnostic tests “based on dimensions of observable behavior” (33) (p. 904).

While a clinical interview remains the basic tool for diagnosing depression (34), linguistic research has demonstrated that systematic analysis of language content reliably classifies patients into appropriate diagnostic groups (35, 36). Nguyen et al. (37) report that computerized word counting techniques (38, 39) discriminate depression communities from other subgroups and also reveal strong online-language predictors of depression (40) and suicide (41). Aberrant written and spoken languages are frequently reported in patients with depression (42–46). Being a chronic affective disorder presenting either within mild depressive symptoms or with marked absence of pleasure in daily activities, dysthymia is characterized by increased speech flow, in contrast to the slowed speech typical of MDD (14). The excessive use of first-person singular pronouns (*I*) correlated with depression in many (22, 23, 38, 46, 47), but not all studies (48). Objective (*me*) and possessive (*my*) first-person pronouns were more frequent in speech of a group with depression, and predicted depression better than did subjective (*I*) pronouns (47). Elevated usage of first-person pronouns was attributed to self-focused attention or self-preoccupation (44, 47, 49). Among various measures of depressive self-focusing style, rumination (repetitions of the same, usually negative, information) has been mentioned in many studies (50–52). Other features of depression included elevated use of mental state verbs (*think*), words denoting causal relations (*because*) (53), greater use of generalizing terms (*everything, always*), negation (*nothing, never*), and words referring to ambivalent emotional states (54, 55). The increased use of discrepancy words (*should)*, possibly reflecting enhanced aspirations for the future (56), has been discussed as a marker of improvement with therapy for depression. Together, these promising results denote that “the styles in which people use words” represent no less meaningful information than “the content of what they say” about their symptoms (38) (p. 548). Nonetheless, language phenomena are still not widely considered for psychiatric diagnosis of affective states.

### Hypotheses

Given this background, we predicted that our exploratory analysis of linguistic variables would reveal a set of word-use patterns for differentiation of MD from NS and euthymic state (see Russian/English examples in Table 1). *Directional hypotheses*. In accord with previous studies on *lexico-grammatical variables* (42), we predicted that MD patients would (1) make excessive use of first-person/personal and other types of indefinite (generalized, negative) pronouns, reflecting words of generalization, negation and ambivalent emotional states revealed in depression (54, 55). *Non-directional hypotheses*. Our specific hypotheses follow: Focusing on *syntactic and lexico-semantic variables*, we explored whether MD patients (2) predominantly used single-clause vs. multi-clause sentences and (3) narration vs. reasoning, as reflecting descriptive vs. analytic thought style. We predicted (4) an increased number of lexical (tautologies) and semantic repetitions in MD as a marker of ruminations and depressive self-focusing style (51, 52), and further explored whether MD (5) favors figurative language (metaphors, similes), and (6) unusual/atypical word order related to their emotionally overwhelmed state (54). Based on some previous studies and our own clinical experience, we also hypothesized that, since ruminations are mostly focused on past negative events, MD patients would express *within lexico-grammatical variables* (7) predominantly with the continuous (the imperfective tense of Russian verbs denoting uncompleted actions) rather than the perfect (perfective type/completed actions) form [state-of-being verbs (32)], and (8) the past rather than present or future tense verbs [past vs. future in depression (57); negative schemas of the past in depression (58)]. Thus, we aimed to apply the linguistic approach as an additional diagnostic key for understanding clinical variability along the continuum of affective states.

**Table 1:** Linguistic variables included in analysis.^a^

Lexico-semantic variables	
Categorical variables	Language type: Narration (*description of facts, states, e.g., «*Сmалu появляться мысли, что я наношу непоправимый психологический вред моему ребенку и мужу*»/“I started having thoughts that I was causing irreparable psychological damage to my child and husband”*) Reasoning (*assessment, causal relations search, e.g., «*Я задавал себе вопрос, зачем мне нужно идти туда и не находил ни одного варианта ответа*»/“I was asking myself: why do I have to go there; and couldn’t find an answer”*)

Quantitative variables	Colloquialisms (*informal words/phrases, e.g., «*не хватает духу*»/“don’t have enough spirit”*) Tautologies (*word and phrases repetitions, e.g., «*делала это, делала это снова и снова*»/“I was doing it, doing it again and again”*) Lexical, semantic repetitions (*e.g., «*плакала и рыдала*»/“I was crying and sobbing”*) Figurative language/rhetorical figures: Metaphors (*figurative comparison, e.g., «*погрязла в этом горе*»/“I am drowning in this grief”*) Similes (*direct comparison, e.g., «*высохла как скелет*»/“I was thin as a skeleton”*)

**Syntactic variables**	

Categorical variables	Predominant sentence type: Single-clause (*e.g., «*Близким от меня одни неприятности*»/“I am just a source of trouble for my family and friends”*) Multi-clause (*e.g., «*Я не думала, что такой купол на меня опустится*»/“I did not think that such a darkness* (*verbatim, cupola*) *would descend upon me”*)
	Single-clause sentence type: Impersonal (*e.g., «*Дальше только хуже*»/“It only gets worse”*) Reduced (*e.g., «*Жизнь-болото*»/“Life is a swamp”*) Complete (*e.g., «*Я просто хотел лежать на диване*»/“I just wanted to lie on the couch”*) Incomplete (*e.g., «*Хочется не проснуться*»/“Want to not wake up”*)
	Multi-clause sentence type: Complex (*absence of causal relations between the clauses’ content within one sentence, e.g., «*В последнее время я думала все чаще, что не нужна никому, никто мной не интересуется*»/“Recently, I have been thinking more and more often, that nobody needs me, nobody cares”*) Compound (presence of causal relations between the clauses’ content within one sentence, *e.g., «*Я постоянно задаю себе вопрос, почему я такой стала*»/“I keep asking myself why I became like this”*)
	Word order: Usual/typical (*correct syntax rules, e.g., «*Я оказалась выброшенной из жизни*»/“I became a throw away from life”*) Unusual/atypical (*e.g., «*жизнь моя стала тяжелой*»/“a life of mine became difficult”*)

Quantitative variables	Unusual/atypical word order/rhetorical figures: Ellipses (*omission of words, e.g., «*он мог делать это, я могла…, тоже*»/“he could do it, I could too”*) Inversions (*unusual/atypical/inverted word order, e.g., «*никогда не чувствовала я так себя*»/“never I have felt this way before”*)

**Lexico-grammatical variables**	

Categorical variables	Person types of pronouns: 1st person singular («я*»/“I”*) or plural («мы*»/“we”*), 2nd person singular («ты*»/“you”*) or plural («Вы*»/“you”*), 3rd person singular («он*»/“he”*) or plural («они*»/“they”*), absence
	Verb tenses types: Continuous (*e.g., «*пыталась*»/“was trying”*), perfect (*e.g., «*сделала*»/“have done”*)Verb tenses:Past (*e.g., «*страдала*»/“was suffering”*), present (*e.g., «*живу*»/“am living”*), future (*e.g., «*закончу*»/“will complete”*)

Quantitative variables	Pronoun types: Indefinite (*e.g., «*что-либо*»/“anything”*), including Generalized (*e.g., «*все*»/“everything”*) and Negative (*e.g., «*никто*»/“nobody”*) Personal (*e.g., «*я*»/“I”*), including possessive (*e.g., «*мое*»/“my”*) and reflexive (*e.g., «*себя*»/“myself”*)

*^a^Examples in Russian and their translation to English are given in brackets*.

## Materials and Methods

### Participants

All 201 subjects gave written informed consent according to the Declaration of Helsinki to participate in the study. The research protocol was approved by the Samara State Medical University’s Ethics Committee in 2009. Patients were examined at the University’s Department of Psychiatry after referral from general practitioners, neurologists, and psychotherapists, and had not previously consulted a psychiatrist or been prescribed psychotropic medications before or during the brief period of investigation. The diagnoses were based on the results of clinical psychiatric interviews delivered by psychiatrists (Daria Smirnova and Gennadii Nosachev) and were coded using ICD-10 diagnostic criteria. Inclusion criteria for patients were (1) 20–60 years of age; (2) Russian as native language; (3) completion of secondary education; (4) absence of psychiatric comorbidities, as defined in the ICD-10 and examined with a clinical psychiatric interview in the University’s Department of Psychiatry, and (5) absence of any overt medical or neurological disorders, based on examination by general practitioners and neurologists upon referral from general practice, or, in the case of patients referred by local psychotherapists, as judged by physicians and neurologists at the University’s Psychiatric Hospital. These criteria yielded 124 patients (group MD: 94 females) of mean (SD) age 42 (12) years, coded according to the following ICD-10 categories: (1) F32.0—mild depressive episode (*n* = 27), (2) F41.2—mixed anxiety and depressive disorder (*n* = 26), (3) F43.20—adjustment disorder, brief depressive reaction (*n* = 29), (4) F43.21—adjustment disorder, prolonged depressive reaction (*n* = 23), or (5) F43.22—adjustment disorder, mixed anxiety, and depressive reaction (*n* = 19). The mean (SD) length of depressive state in MD cases was 40 (13) days. Most MD had a college or university degree (*n* = 66; 53%) and lived in an urban area (*n* = 96; 77%). During clinical interview, patients responded to the question about their life problems or stressors according to the categorization of potential life hazards presented in the rubric Z of ICD-10. The majority of patients (*n* = 63; 51%) mentioned problems with their primary social group, including family circumstances, 30 (24%), social environment, 22 (18%), employment and unemployment, and 9 (7%), housing and economic circumstances.

Healthy controls (HC), including subgroups of normal healthy (NH) and individuals in a state of NS, were recruited from among volunteers invited by public announcement and signage. Each HC participant was interviewed separately by two psychiatrists (Daria Smirnova and Gennadii Nosachev) of the University’s Department of Psychiatry to confirm an absence of history of mental disorders in the past and any present diagnoses based on ICD-10 diagnostic criteria. Qualification of NS state in HC participants was consensus-based (Daria Smirnova and Gennadii Nosachev). Inter-rater reliability on categorization of NH vs. NS between two psychiatrists was high: *k* = 0.894, *p* < 0.001, 95% CI (0.795–0.993). HC included 77 age- and education-matched native Russian speakers (61 females) of mean (SD) age 40 (12) years. Among HC, 42 participants were designated as NH and 35 were qualified as being in a state of normal sadness (NS), based on reporting current life problems and low mood. The NS individuals were coded as having potential health hazards according to the following ICD-10 categories: Z56—problems related to employment and unemployment (*n* = 7), Z59—housing and economic circumstances (*n* = 14), Z60—social environment (*n* = 4), and Z63—primary support group, including family circumstances (*n* = 10).

### Data Collection Procedures

Clinical psychiatric interviews were used as a database for psychopathological evaluation. In the psycholinguistic approach, we focused on the written self-reports [on the topic (i) “The current state of life and future expectations” and (ii) “The meaning of life”] provided by all participants. The instruction on each of two topics was given orally by a researcher as follows: “Please write as much as you think is necessary and take as much time as you need to describe your current state of life and future expectations.” In total, 402 texts were analyzed by the research team, which included a psychiatrist (Daria Smirnova), linguist (Elena Sloeva), and clinical psychologist (Natalia Kuvshinova). While one rater (Daria Smirnova) was necessarily informed about the clinical state of the individuals (patients or HC), the other two raters were blind regarding the group assignment. Both blind raters analyzed the entire sample regarding linguistic variables. The HDRS (21 items) validated Russian version was administered to all subjects. HDRS raters were not blind to MD group, as patients had been referred with the preliminary diagnosis of depression. As for the HC group, HDRS scores have been recorded before the HC (NH vs. NS) group allocation.

### Psycholinguistic Analysis

Written samples were analyzed with respect to the number of words in the text using MS Word properties and hand-coding procedures: (i) lexico-semantic [e.g., rhetorical figures (metaphors, similes)], (ii) syntactic [e.g., predominant sentence type (single-clause, multi-clause)], and (iii) lexico-grammatical [e.g., pronouns (indefinite, personal)]. We defined categorical variables according to the participant’s predominant usage of each relevant linguistic unit in each linguistic sample. For example, if a participant used 5 single-clause sentences and 10 multi-clause sentences, then the estimate of the variable “Predominant sentence type” was specified as “multi-clause.” Quantitative variables were scored as quotients according to the number of the relevant units over a span of 10 sentences. In other words, if a participant used 6 metaphors across 20 sentences, then the quotient of metaphors is equal to 3, calculated as the proportion per 10 sentences. All the variables are summarized in Table 1.

### Statistical Data Analysis

All data were checked for the assumption of normality using the Shapiro–Wilk test and by inspection of histograms. Differences between study groups were calculated using the non-parametric Mann–Whitney *U*-test, two-tailed, Pearson chi-square test, and one-way ANOVA, depending on the type of variables and number of groups compared. Spearman’s bivariate and point-biserial correlations were used to analyze relationships between linguistic data and demographic variable of gender. Values of *p* < 0.05 were considered statistically significant. An inter-rater reliability analysis using the Cohen’s kappa statistic was performed to determine consistency between raters on categorization of NH and NS groups and on linguistic variables. Discriminant analysis (Wilks’ λ) was used to establish the level of significance in relation to diagnostic types based on linguistic variables. All statistical analyses were performed with the IBM SPSS Statistics 22 (59).

## Results

### Clinical Description of MD and NS

From the psychiatrist’s clinical perspective using the classical approach of descriptive psychopathology, a state of MD was characterized by the following signs and symptoms: (i) depressed mood, consisting of sadness, sorrow, irritability, despondency, or melancholy, (ii) mood swings during the day with predominant hypothymia, and (iii) more prominent mood changes in reaction to current life events. The depressive condition affected the patient’s quality of life and was perceived by the patient as a pattern of unwanted or even alien behavioral reactions. Furthermore, MD included partial anhedonia and distortion of self-image to reflect low self-esteem, lack of self-confidence, and self-dislike. Patients also expressed difficulties in decision-making, as well as a pessimistic perception of current life events. Their complaints included a negative view of the past, with emphasis on committed mistakes and failures. Finally, MD was associated with loss of energy, fatigue and lack of interest in social activities. Their somato-autonomic dysfunction manifested in sleep disturbances, changes in appetite, reduced libido, and asthenia.

In contrast to MD, self-perception in the NS subgroup was expressed as an adequate and appropriate reaction to current adverse life events. While NS participants described their emotional experience as a constant subjective feeling of dissatisfaction regarding objective life circumstances arising from external reasons, their ideation was focused on the details of their problematic life situation. The NS group continued their usual daily activities, but with some muting of interests and periods of ruminations accompanied by feelings of sadness. The NS further differed from MD in their focus on present difficulties while analyzing their decision-making and problem-solving strategies, and in that they commonly described future aspirations.

### Psychometric Measures

In the MD group, the mean (SD) HDRS-21 total score was 14.3 (2.20), which differed significantly from the NH and NS subgroups: HC-3.03 (0.89), NS-3.77 (0.65), NH-2.40 (0.50), using ANOVA *F*(2, 198) = 4,110.05, *p* < 0.001, η^2^ = 0.976, and with significant paired between-groups differences found using *post hoc* Bonferroni correction (*p* < 0.05; α = 0.05), *p* < 0.001.

### Linguistic Features of MD as Compared with NS and Euthymic State

Mild depression patients produced longer written responses than HC (including NS and NH); the mean (SD) number of words per text was 311 (58) for MD vs. 209 (42) for NS and 197 (29) for HC, *F*(107, 93) = 4.17, *p* < 0.001, effect size η^2^ = 0.827. Written language of MD patients demonstrated distinct peculiarities. The effect sizes were intermediate or large for most differing variables of language (Tables 2 and 3). No significant and/or strong correlations were observed between linguistic variables and the factor of gender (all *p* > 0.05). The average inter-rater reliability on linguistic variables between two blind raters was high: *k* = 0.840, *p* < 0.001, 95% CI (0.807–0.865).

**Table 2:** Lexico-semantic and syntactic features in MD, NS, and healthy individuals.

Linguistic variables (quotients)		Descriptive statistics					Between-group comparisons			

		Statistical variables	MD (*n* = 124)	Control group			MD vs. HC*		MD vs. NH vs. NS*	
					
				HC (*n* = 77)	Subgroups		Mann–Whitney *U*-test		One-way ANOVA	
					
					NH (*n* = 42)	NS (*n* = 35)				
								
							*U*	Effect size *r*	*F* df (2, 198)	Effect size η^2^
Colloquialisms (informal words/phrases)		Mean SD	3.74 0.44	1.21 0.47	1.02 0.15	1.43 0.61	1,675.50	0.631	36.84	0.271

Tautologies (words/lexical repetitions)		Mean SD	3.77 0.42	1.44 0.50	1.26 0.45	1.66 0.48	2,604.00	0.503	7.18	0.067

Lexical and semantic repetitions		Mean SD	4.42 0.50	1.82 0.39	1.69 0.47	1.97 0.17	2,394.00	0.445	30.93	0.238

Rhetorical figures	Ellipses (omission of words)	Mean SD	1.91 0.29	1.53 0.53	1.38 0.54	1.71 0.46	0.00	0.253	518.91	0.409
	Inversions (unusual word order)	Mean SD	4.00 0.00	1.08 0.27	1.00 0.00	1.17 0.38	923.50	0.934	104.49	0.839
	Metaphors (figurative comparison)	Mean SD	2.55 0.84	1.40 0.83	1.48 0.86	1.31 0.80	3,534.00	0.712	68.62	0.513
	Similes (direct comparison)	Mean SD	1.91 0.29	1.53 0.53	1.38 0.54	1.71 0.46	3,449.00	0.261	18.13	0.156

*p < 0.05.

*HC, the entire healthy control group; NH, normal healthy participants with euthymic state; NS, normal sadness; MD, patients with mild depression*.

**Table 3:** Pronouns use in MD, NS, and healthy individuals.

Linguistic variables (quotients)		Descriptive statistics					Between-group comparisons			

		Statistical variables	MD (*n* = 124)	Control group			MD vs. HC*		MD vs. NH vs. NS*	
					
				HC (*n* = 77)	Subgroups		Mann–Whitney *U*-test		One-way ANOVA	
									
					NH (*n* = 42)	NS (*n* = 35)				
								
							*U*	Effect size *r*	*F* df (2, 198)	Effect size η^2^
Pronouns	Indefinite (e.g., *anything*)	Mean	3.75	1.40	1.29	1.54	129.50	0.916	67.85	0.406
		SD	0.52	0.54	0.46	0.61				
	Generalized (e.g., *everything*)	Mean	3.74	1.58	1.46	1.70	663.50	0.860	4.06	0.039
		SD	0.52	0.60	0.52	0.64				
	Negative (e.g., *nobody*)	Mean	3.75	1.40	1.29	1.54	94.50	0.932	482.81	0.829
		SD	0.51	0.50	0.41	0.60				

	Personal (e.g., *I*)	Mean	3.45	1.83	1.83	1.83	158.00	0.842	235.24	0.704
		SD	0.52	0.52	0.49	0.57				
	Possessive (e.g., *my*)	Mean	3.75	1.58	1.45	1.74	600.00	0.782	153.13	0.607
		SD	0.50	0.61	0.59	0.61				
	Reflexive (e.g., *myself*)	Mean	3.56	1.96	1.90	2.03	1,364.00	0.697	390.95	0.797
		SD	0.54	0.50	0.58	0.38				

*p < 0.05.

*HC, the entire healthy control group; NH, normal healthy participants with euthymic state; NS, normal sadness; MD, patients with mild depression*.

#### Lexico-Semantic Variables

Responses in MD patients, compared with those in HC, were organized more often as narration (MD: 106/124; 85%; HC: 55/77; 71%) and less often as reasoning (MD: 18/124, 15%; HC: 22/77, 29%), χ^2^(1) = 5.89, *p* = 0.015, effect size *w* = 0.171, i.e., more often in a descriptive rather than analytic manner. The NH employed more utterances based on reasoning (12/42; 29%) than MD patients (18/124; 15%), χ^2^(1) = 4.19, *p* = 0.041, *w* = −0.159.

Mild depression patients used more colloquialisms or informal words/phrases (Table 2). Responses in MD also had more repetitions, both with respect to re-using the same words (tautologies) and to expressing the same idea multiple times (lexical and semantic repetitions). The MD group used significantly more metaphors and similes (figurative language) than HC (Table 2). In comparison with euthymic NH, the NS group was impoverished at the lexico-semantic sublevel, showing greater use of tautologies and repetitions, in general (Table 2).

#### Syntactic Variables

Ninety nine (80%) MD, compared with only two (2.6%) HC individuals, predominately used single-clause sentences, χ^2^(1) = 113.37, *p* < 0.001, *w* = 0.751. Among single-clause sentences, reduced sentences appeared and often predominated in 73% of MD (*n* = 91), compared with 17% of HC cases (*n* = 13), χ^2^(5) = 141.34, *p* < 0.001, *w* = 0.839. Among multi-clause sentences, compound sentences were predominately used over complex sentences by the majority of the MD group (106/124, 85%), and more often than in HC (56/77, 73%), χ^2^(1) = 5.7, *p* = 0.017, *w* = 0.168. A predominant atypical/inverse word-order usage was also revealed in patients [MD: 124, 100%; HC: 5, 6.5%; χ^2^(1) = 180.66, *p* < 0.001, *w* = −0.948]. NS used atypical word-order forms (ellipses and inversions) more often than participants in euthymic state (Table 2). There were no significant findings regarding the preferences in the sentence-type use in NS as compared with either NH or MD.

#### Lexico-Grammatical Variables

Patients’ responses contained significantly more personal and indefinite pronouns, compared with NS and HC (Table 3). Data showed greater usage specifically of first-person singular pronouns (e.g., *I, me, my*) in MD (124/124, 100%) than in HC (55/77, 71%), χ^2^(3) = 39.78, *p* < 0.001, *w* = 0.445. MD patients predominately used verbs in continuous tense [MD: 116/124, 94%; HC: 26/77, 34%; χ^2^(1) = 81.87, *p* < 0.001, *w* = 0.638] and in past tense [MD: 124/124, 100%; HC: 2/77, 3%; χ^2^(2) = 192.67, *p* < 0.001, *w* = 0.979], mostly in first-person singular and impersonal forms [MD: 104/124, 84%; HC: 6/77, 8%; χ^2^(5) = 69.38, *p* < 0.001, *w* = 0.588]. While MD used more continuous verbs in the past tense, significantly and with large effect size as shown above, HC used perfect verbs (51/77, 66%) and verbs in the present (51/77, 66%) and future tense (24/77; 32%). Language in NS, compared with NH, included more verbs in continuous form [NS: 19/35, 54%: NH: 7/42, 17%; χ^2^(1) = 12.08, *p* = 0.001, *w* = 0.396] and in the present tense [NS: 31/35, 89%; NH: 20/42, 48%; χ^2^(1) = 20.57, *p* < 0.001, *w* = 0.517].

### Mathematical Modeling of Diagnostic Types of MD, NS, and Euthymic State

**Figure 1:**
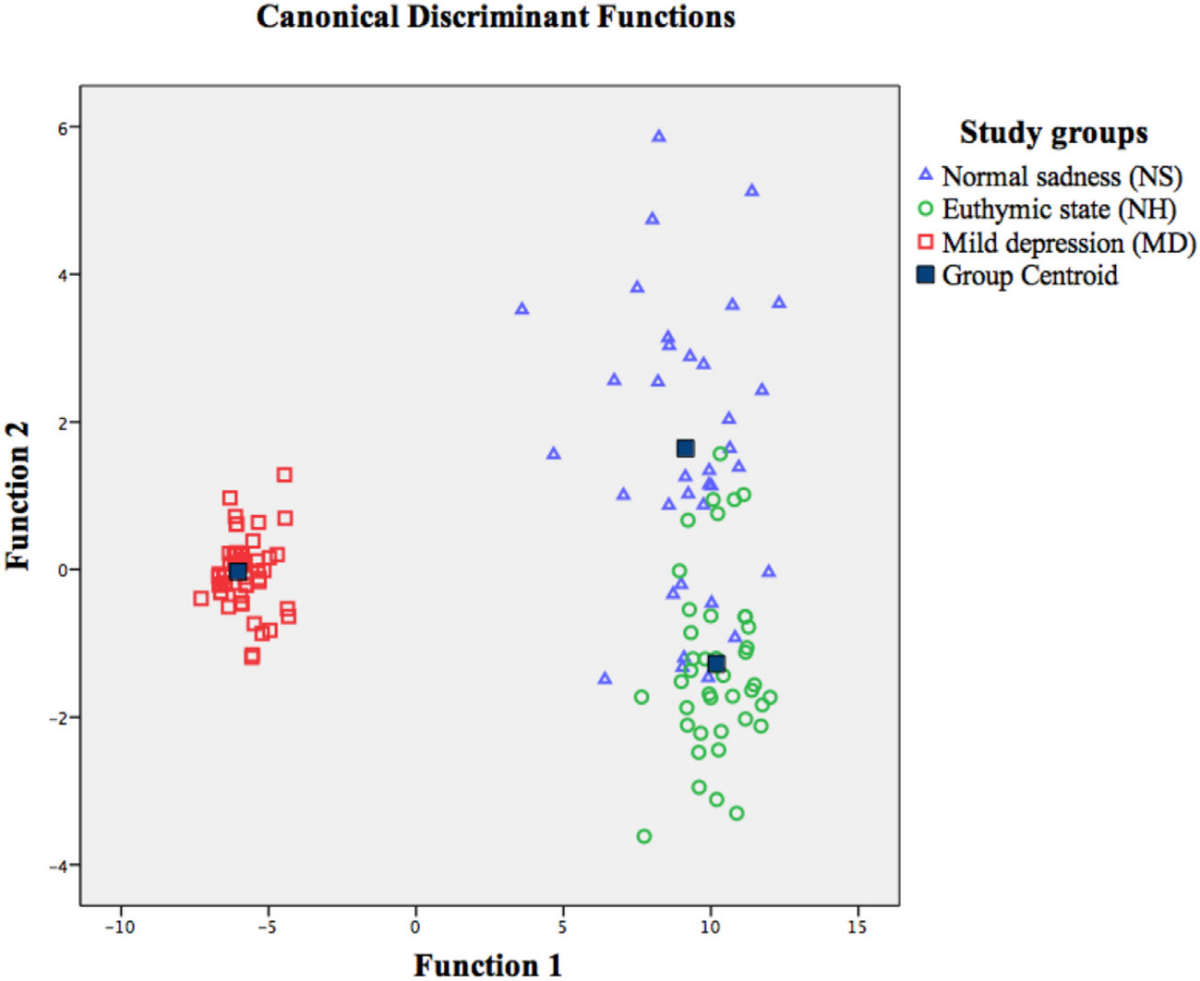
Discriminant model of the diagnostic types of mild depression, normal sadness, and euthymic state in healthy participants, based on linguistic variables (excluding sentence-type analysis).

Discriminant analysis was performed to establish the level of distinction in linguistic features between investigated study groups. The elements of diagnostic types MD, NS, and NH included lexico-semantic, syntactic, and lexico-grammatical variables, excluding the sentence-type indicators, which did not show significant differences in the between-group analysis for NS (Table 1). The model was elaborated using standard SPSS methods to generate a linear equation for calculation of discriminant tabs, as well as validation and refinement of the model’s adequacy. Integrated analysis of the discriminant functions revealed the high congruity in classification. 92.5% of original and 89.6% cross-validated grouped cases were correctly classified. The analysis results confirm that our discriminant model significantly characterizes the study sample such that the set of linguistic variables discriminates the states of MD, NS, and euthymic state in NH. The spread of the canonical values in the discriminant model reveals significant differences between MD, NS, and NH [98.6%; test of function 1 through 2: Wilks’ λ(40) = 0.009, *p* < 0.001, canonical correlation *r* = 0.992]. The structure matrix of discriminant analysis demonstrated that the highest significant predictors for classification of the three groups were the following variables: (i) ellipses (*r* = 0.583), (ii) colloquialisms (*r* = 0.534), (iii) the verb tense (*r* = −0.460), (iv) negative pronouns (*r* = 0.451), (v) word order (*r* = −0.405), (vi) verbs form (*r* = 0.345), and (vii) reflexive pronouns (*r* = 0.344). Based on these data, we repeated the discriminant analysis using only variables with the highest predictability, which yielded similar results [98.3%; test of function 1 through 2: Wilks’ λ(14) = 0.015, *p* < 0.001, *r* = 0.987] (Figure 1). As shown in Figure 1, MD stands out from NS and NH by function 1, and the centroids for all three groups are significantly different. Collinearity statistical analysis revealed that the variables of verb tense (Tolerance = 0.079, VIF = 12.736) and word order (Tolerance = 0.075, VIF = 13.335) may be responsible for the multicollinearity. However, the discriminant analysis excluding these variables demonstrated highly significant differentiation of diagnostic types MD, NS, and NH based on the remaining language indicators [97.2%; test of function 1 through 2: Wilks’ λ(34) = 0.020, *p* < 0.001, *r* = 0.982]. To specify the contribution of affective component on language use, we also performed another exploratory analysis including the subgroups of MD with and without anxious features, NS, and euthymic state [97.4%; test of function 1 through 3: Wilks’ λ(57) = 0.007, *p* < 0.001, *r* = 0.990] (Data Sheet S1 in Supplementary Material).

## Discussion

By choosing the topics for written reports for patients, we intended the diagnostically relevant mental state to appear in the written speech, thus matching responses to the clinical interview and reflecting the context of past and present in the frame of the patients’ description of their depressed mood. We assigned the topics about future expectations and meaning of life to document the patients’ positive resources, motivations, and potential ability to use the context of the future as reflecting these perspectives for future recovery. However, we concede that these topics might have biased the emotional involvement in patients and thus influenced the content of written reports, as well as the writing style. Our study demonstrated that language of MD patients was characterized by significant differences within the set of lexico-semantic, syntactic, and lexico-grammatical variables, as earlier shown within some language indicators for depression (32, 43, 44, 46). In agreement with a report of increased speech flow in dysthymia, which is mostly characterized by mild depressive symptoms with a chronic course (14), as distinct from the briefer responses in MDD (60), we found longer written responses emerged as a diagnostic sign for discrimination of MD and HC. As predicted, while providing longer responses, our MD patients predominantly used single-clause sentences, reduced utterances, and incomplete phrases with omission of words (ellipses), which reflects the language flow interruptions previously observed in studies of clinical depression (45, 60, 61). We suppose that the pattern of frequent usage of rhetorical figures within phenomena of figurative language (metaphors, similes) and atypical word order (inversions, ellipses) in MD could be interpreted as arising from overt emotional dominance in language content, following the concept presented by Pennebaker et al. (38) about language features reflecting emotional states and self-perception. According to language development theories (62), figures of speech/rhetorical figures are acquired early in age and their increased use may point to a regression toward earlier forms of language.

Our finding of increased usage of typically oral language expressions (colloquialisms), which was among the highest predictors for differentiation between MD, NS, and NH, together with unusual/atypical word order, confirmed the hypothesized predomination of conversational style over standard written language patterns in MD. Patients seemingly had a certain lack of flexibility, such they could not readily shift from oral conversation with the researcher into the written style appropriate for the self-reporting task. This resembles their difficulty in switching from depressive self-focused attention and ruminations (lexical/word and semantic/topic repetitions) toward potential positive thinking and adaptive coping strategies (50–52).

We also established that, within multi-clause sentences, our MD patients more often used compound sentences (without causal relations between the clauses content with a sentence) than complex sentences (with causal relations between the clauses). This finding in MD stands somewhat in contrast to that of Pennebaker et al. (53), who found generally increased use of causation words (typical for compound-type rather than complex-type multi-clause sentences) in depression, although we did not explicitly rate causation words. In combination with the finding of a predominant use of the single-clause sentences, these properties of sentence use revealed a more frequent addressing to descriptive rather than analytic thought strategies. From a developmental point of view (62), descriptive strategies within narration may represent an early acquired or basal form of verbal behavior, in comparison with the mature analytic style within reasoning acquired later in life. This scenario suggests that MD entails regression in the style of using verbal strategies for organizing the discourse (62). While HC used a mature strategy, including both analysis of events and intellectual reflection (self-analysis and problem-solving behavior), intellectual reflection in MD was subsumed by a sensual/emotional reflection within passive narration.

Consistent with previous findings on greater pronoun use within the context of depressive self-focusing or self-preoccupation style (38, 44, 46, 47, 49, 52), we found that an increased number of personal (e.g., *I*), possessive (e.g., *my*), reflexive (e.g., *myself*) pronouns, gave significant discrimination of MD, NS, and NH. Higher use of personal pronouns was earlier described for healthy participants of female gender (63), but this was not evident in our sample. Enlarged use of generalized (e.g., *everything*) and negative (e.g., *nobody*) indefinite pronouns confirmed previously obtained data describing the overt emotional dominance within generalization, negation, and polarity in emotional expression in depression (54, 55). Frequent use of negative pronouns may refer to the coping mechanisms of denial and negation associated with depressive symptoms or depressive personality traits (44). Insofar as pronouns lack semantic content in their word root, we suggest that their increased use in MD conveys loss of specific meanings in speech and could also be interpreted as a manifestation of semantic impoverishment; this is in keeping with data on reduced semantics in depression (64) and mild cognitive impairment (65).

As we hypothesized, written language in MD was shifted into the past, reflected not only through ruminations about past life events within lexical and semantic repetitions but also in the increased frequency of past tense verbs (57, 58). This concurs with studies demonstrating that depressed patients use fewer discrepancy words (e.g., *should*), which typically symbolize aspirations for the future (56, 66). Our patients used more verbs in continuous/imperfective form, as earlier noted by Andreasen and Pfohl (32). The self-perception of time in MD within the past tense verbs emerged as an additional discriminative feature of the high predictability for differentiation of affective states in our study.

Our findings regarding the patterns of language use as a result of affect or mood influence may reflect not only symptomatic behavior and thinking within the affective states of MD or NS but could also be indicative of stable personality traits or defensive mechanisms, a possibility that requires further investigation (44). However, our discriminant model significantly differentiated the conditions of MD from NS and euthymia with a probability of 98.6%. Another discriminant model using linguistic indicators significantly differentiated the states of MD with and without anxious features, NS and euthymia with the similar level of probability (97.6%). These data may support our hypothesis about the particular effect of affective component on the deviations in language use. This result, which confirms and extends the observations in depression by Oxman et al. (35), Desmet and Hoste (67), Kahn et al. (68), and others, also illuminates the role of assessment of verbal behavior in MD and NS for clarifying the continuum and variety of affective states.

### Limitations of the Study and Implications for Further Research

We analyzed only written texts but did not record examples of natural oral speech flow. We used hand-coding procedures and did not apply the Linguistic Inquiry and Word Count (39) computer program elaborated to categorize the text into linguistic categories, because this does not yet exist for Russian language. Also, given the large number of variables examined in the study, we must consider the possible occurrence of type I errors related to interpretation of results. As no patients with psychiatric comorbidities were included, we accordingly isolated the influence of depressive affect on language. As such, we do not take a strong position related to the generalizability of findings in our sample but propose a broader investigation addressing these potential confounds. Future studies might benefit from examining the relationships between language patterns in patients with affective states and their personality traits, thus aiming to define the contribution of personality factors on language use.

We expect that these results will draw more attention to the diagnostic significance of language assessment in psychiatry and clinical disciplines and show that verbal behavior is a sensitive diagnostic marker in MD. We also suggest that this would encourage practitioners to attend not only to *what* the patient utters but also *how* it is spoken. There remains a need for more data regarding linguistic features of conversational language in depression and for generalization to different languages, so as to support a broader applicability of the concept of diagnostic criteria based on written language, and to support precise recommendations for guidelines in clinical practice. In relation to practical implementation, for example, these results might inform the development of a standard questionnaire for diagnosis of MD through written language patterns, designed to be administered by non-experts, and perhaps automatically scored. Present results lead us to contend that linguistic study could inform future clinical approaches to non-pharmacological treatment of MD. Such psychotherapeutic approaches would address not only language content but also language remediation or cognitive training of language style and structure. If symptoms are indeed partially organized by language structure, a treatment approach to normalizing of language might play a beneficial role in improving affective state.

## Ethics Statement

All subjects gave written informed consent according to the Declaration of Helsinki to participate in the study. The research protocol was approved by the Samara State Medical University’s Ethics Committee.

## Author Contributions

DS, GN, and ES designed the project. DS and GN collected the data. DS, GN, ES, and NK analyzed the data with advice from DR and PC. DS, GN, and PC wrote the first draft of the manuscript. All the authors reviewed the final version of the manuscript.

## Conflict of Interest Statement

The authors declare that the research was conducted in the absence of any commercial or financial relationships that could be construed as a potential conflict of interest. The reviewer RS and handling editor declared their shared affiliation.
