# Supplementary material for: Language Patterns Discriminate Mild Depression From Normal Sadness and Euthymic State
Source: Front Psychiatry. 2018 Apr 10;9:105. doi: 10.3389/fpsyt.2018.00105 (PMC5902561; doi:10.3389/fpsyt.2018.00105)
Supplement: Supplementary file 1 [file Data_Sheet_1.DOCX]

**Supplementary material.**

**Discriminant model of the diagnostic types of mild depression with and without anxious features, normal sadness and euthymic state, based on linguistic variables**

The MD group included the subgroup of MD with prominent features of anxiety (n=45; 36%). The affective component of depression was characterized by a higher level of anxiety symptoms within the HDRS Total score (Mean=14.80, SD=0.73) and HDRS Anxiety Psychic score (Mean=2.42, SD=0.87), as compared to the subgroup of MD without anxious features (n=79; 64%) (HDRS Total score: Mean=14.01, SD=1.02, t(122)=-4.56, p<0.001, d=-0.83; Anxiety Psychic score: Mean=0.94, SD=0.49, t(122)=-12.22, p<0.001, d=-2.21). Discriminant analysis was performed to establish the level of distinction in language patterns between MD subgroups with and without anxious features, and NS and NH subgroups based on lexico-semantic, syntactic and lexico-grammatical variables. The spread of the canonical values in the discriminant model revealed significant differences between the investigated subgroups, pointing to relationships between the affective component and patterns of language use (97.4%; test of function 1 through 3: λ–Wilks(57)=0.007, p<0.001, r=0.990) (Figure).


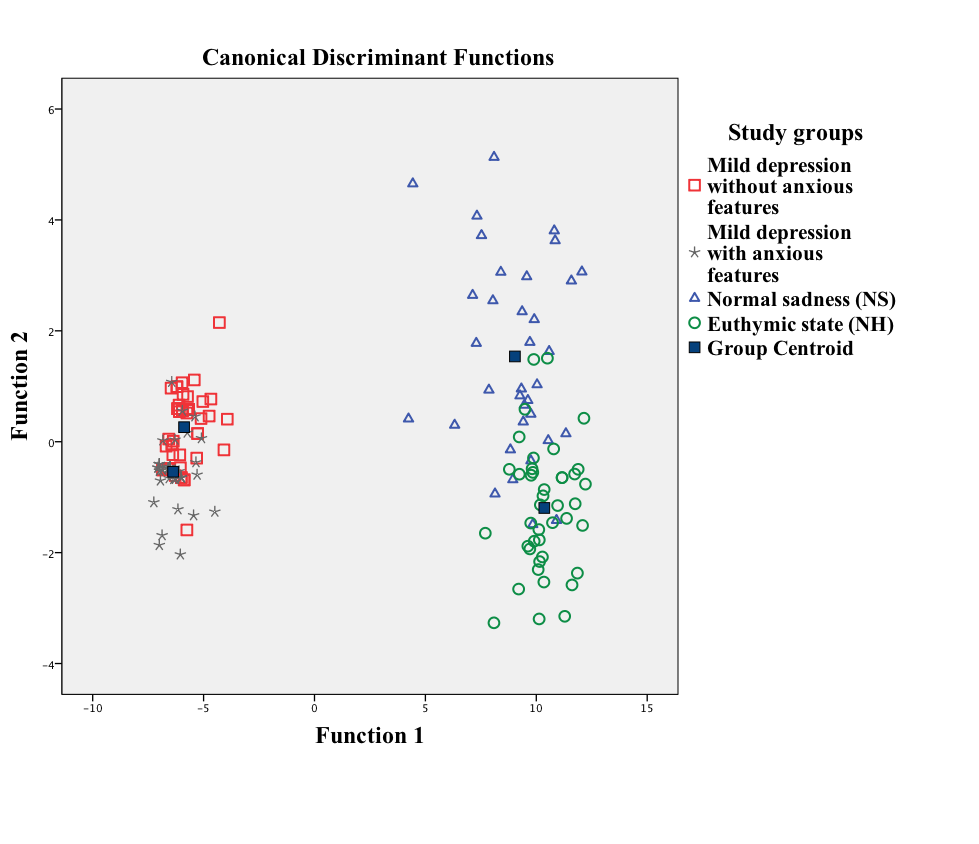


**Figure. Discriminant model of the diagnostic types of mild depression with and without anxious features, normal sadness and euthymic state in healthy participants, based on linguistic variables**
